# Prevalence and Determinants of Frailty in Community‐Dwelling Iranian Older Adults: A Cross‐Sectional Study

**DOI:** 10.1002/hsr2.71202

**Published:** 2025-09-01

**Authors:** Majid Rahimi, Maryam Shirdozham, Awat Feizi

**Affiliations:** ^1^ Department of Health Education and Health Promotion, School of Health Isfahan University of Medical Sciences Isfahan Iran; ^2^ Department of Epidemiology & Biostatistics, School of Public Health, and Isfahan Clinical Toxicology Research Center Isfahan University of Medical Sciences Isfahan Iran

**Keywords:** determinants, elderly people, frailty, polypharmacy, retirement

## Abstract

**Background and Aims:**

Frailty is one of the most common syndromes in old age. This syndrome, associated with adverse health outcomes and increased economic costs, can affect the quality of life of elderly people. This study investigates the prevalence and determinants of frailty in community‐dwelling Iranian older adults.

**Methods:**

This analytical cross‐sectional study was conducted over 5 months among the retired elderly people (> 60 years) in Isfahan. The tools used in this study included a demographic questionnaire, the Pittsburgh Sleep Quality Index (PSQI), an assessment of polypharmacy, and the Edmonton Frail Scale. Data were analyzed using statistical tests in SPSS software.

**Results:**

The prevalence of frailty, based on the Edmonton Frail Scale, was 17.4%. No statistically significant difference in mean frailty scores was observed between men and women (*p* = 0.286). Logistic regression analysis showed that older age (*B* = 0.067, OR = 1.07, 95% CI: 1.01–1.15), lower education (*B* = −2.66, OR for MSc = 0.07, 95% CI: 0.00–0.74), polypharmacy (*B* = 1.82, OR = 6.16, 95% CI: 2.8–13.50), and poor sleep quality (*B* = 0.59, OR = 1.80, 95% CI: 0.88–3.78) were significantly associated with a higher likelihood of frailty (*p* < 0.05).

**Conclusion:**

This study's results indicated that age, education, polypharmacy, and poor sleep quality are associated with an increased risk of frailty in Iranian elderly people. Therefore, timely screening and intervention are recommended to identify these factors and prevent their irreversible physical, psychological, and financial consequences.

## 1 Introduction

The global increase in the elderly population, due to declining mortality rates and rising life expectancy, has become a worldwide phenomenon [1]. The World Health Organization (WHO) has predicted that the proportion of individuals over 60 will increase from 12% in 2015 to 22% by 2050 [2]. In Iran, the national census indicated that 9.3% of the population was aged 60 and above [3]. Projections suggest this figure will rise to approximately 32% by 2051 [4, 5].

Given the growing elderly population, frailty has emerged as one of the most common geriatric syndromes, garnering significant attention from geriatric specialists [6]. Frailty is a cumulative decline in the body's physiological reserves and functions. It becomes under stress events and increases the risk of adverse outcomes [7]. In old age, frailty is often a precursor to adverse clinical events, reflecting chronic health problems and the medical needs of elderly people [8]. Several factors, including genetics, hormonal changes, and inflammation, are considered key contributors to the development of frailty in elderly people [9]. The prevalence of frailty is around 6%–11% among community‐dwelling elderly people and between 25% and 65% among hospitalized elderly patients [10]. Frailty in older adults increases the risk of falls, disability due to accidents, hospitalization, and mortality [11]. A study conducted among the Iranian elderly people found that the prevalence of frailty was 33.4%, and the associated factors included older age, female gender, lower education level, lower income satisfaction, marital status (being single), and living alone [12].

Various chronic diseases contribute to frailty [13]. Poor sleep quality is one clinical condition that places older adults at greater risk for frailty [14]. Many elderly individuals complain of age‐related changes in sleep quality [15], with more than 50% of people over the age of 65 experiencing sleep disorders [16]. A study among older men found that poor sleep quality was associated with a higher likelihood of frailty and mortality [17]. Additionally, an Iranian study reported that 42.7% of participants had poor sleep quality, and there was a direct relationship between poor sleep quality and frailty in elderly people [18].

As people age, they often develop multiple chronic conditions that require multiple medications, leading to polypharmacy [19]. This increases vulnerability and mortality among the elderly [20]. Frailty and polypharmacy have a bidirectional relationship, with frailty influencing drug effects [21].

Retirement, on the other hand, is a distinct stage of life that significantly impacts the daily activities and social interactions of elderly people [22, 23]. For some, retirement is accompanied by frailty and the need for care from others [24]. Retirement can transform a healthy adult into a vulnerable individual with diminished physiological capacities and increased susceptibility to various diseases and death [25]. Therefore, identifying and planning to mitigate the problems of elderly people and retirees can improve their health and reduce economic costs for both families and the government [26].

With increased life expectancy and the aging population, the proportion of elderly retirees with health issues is also rising. However, there is limited information on the current prevalence of frailty among retired elderly individuals in Iran. To date, the information related to factors affecting frailty in Iranian retired elderly people is insufficient. This study investigates the assessment of Frailty and Its Related Factors among retired elderly individuals in Isfahan, Iran.

## 2 Methods

### 2.1 Study Design

To investigate the prevalence of and factors associated with frailty among retired Iranian elderly, an analytical cross‐sectional study was conducted. Data for this study were collected over a 5‐month period, from April 20, 2024, to September 21, 2024, at the Retirement Association of the Education Department in the city of Isfahan, Iran.

### 2.2 Ethical Consideration

The study received approval from the Isfahan University of Medical Sciences Ethics Committee (Research Proposal Code: 1401336 and Ethical Code: IR.MUI.RESEARCH.REC.1402.055). Informed consent was obtained from all study participants. All steps of the study were conducted in strict adherence to the ethical standards of the Helsinki Declaration. We also followed the CONSORT guidelines (http://www.consort-statement.org/) for reporting clinical trials, ensuring that all relevant guidelines and regulations were followed in all methods.

### 2.3 Consent

After explaining the purpose and the nature of the study, informed consent was obtained from the participants, and they had the right to refuse to fill it out. Anonymity and confidentiality of participants were ensured by the authors.

### 2.4 Study Population and Setting

The study population included all elderly members of the Retirement Association of the Education Department in Isfahan (Retirees aged 60 and over). The inclusion criteria for this study were: age 60 years or older, residence in the city of Isfahan, no self‐reported psychiatric illnesses, no severe hearing or vision problems, no cancer or history of debilitating diseases such as severe heart failure or stroke, as well as Parkinson's disease, and no musculoskeletal disorders, and willingness to participate Exclusion criteria included incomplete completion of questionnaires and lack of cooperation at any stage of the research.

### 2.5 Sampling Strategy and Sample Size Calculation

The data were collected using simple random sampling. The sample size was estimated to be 258 participants, considering a significance level of 0.05, a statistical power of 90%, and a correlation coefficient (*r* = 0.2) [27] between polypharmacy and frailty.

### 2.6 Study Instruments

#### 2.6.1 Demographic Information Questionnaire

It assesses age, gender, marital status, education level, family composition, housing ownership status, and the number of children.

#### 2.6.2 Pittsburgh Sleep Quality Index (PSQI)

This sleep quality questionnaire was developed by Buysse and colleagues in 1989 [28]. The questionnaire contains 9 questions, but since the fifth question has 10 sub‐questions, the questionnaire comprises 19 questions in total. It is scored on a 4‐point Likert scale ranging from 0 to 3. The questionnaire includes 7 subscales: subjective sleep quality, sleep latency, sleep duration, sleep efficiency, sleep disturbances, use of sleep medication, and daytime dysfunction [29]. Each component can receive a maximum score of 3. The total score of the tool is formed by the sum of the average scores of these 7 components, with a range from 0 to 21. The higher the score obtained, the lower the quality of sleep, and a score above 6 indicates poor sleep quality. The internal consistency of the questionnaire developed by Buysse and colleagues was calculated using Cronbach's alpha at 0.83. In the Iranian version of the questionnaire, the validity was 0.86 and the reliability was 0.89 [30]. In Zeinalpour's study, the reliability of the tool, using Cronbach's alpha for the entire questionnaire, was found to be 0.74 [31].

#### 2.6.3 Edmonton Frail Scale (EFS)

This questionnaire is a vulnerability assessment tool that can be applied to various populations and was developed by Rolfson and colleagues. Its internal consistency was measured using Cronbach's alpha at 0.62 [32]. The questionnaire includes 9 domains and 11 items, with a total score ranging from 0 to 17. A higher score indicates a higher level of vulnerability. One of the advantages of this tool is its ability to assess various dimensions of vulnerability. The scoring levels categorize individuals as non‐frail with scores below 5, apparently vulnerable with scores of 6–11, and frail with scores of 12–17 [33]. The validity and reliability of the Edmonton Frail Scale in the study by Hosseini and colleagues were confirmed with qualitative face validity and a reliability score of 0.87 using Cronbach's alpha [34].

#### 2.6.4 Polypharmacy Assessment

For polypharmacy assessment, participants were asked to report the number of medications they were taking, and elderly individuals taking more than five medications were considered to have polypharmacy.

### 2.7 Statistical Analysis

Descriptive statistics were used to summarize the demographic characteristics of the study participants. Categorical variables were presented as frequencies and percentages, while continuous variables were expressed as mean ± standard deviation.

To compare determinants of frailty between frail and non‐frail individuals, appropriate statistical tests were used based on variable type. The independent *t*‐test was employed for continuous variables, and the *χ* ^2^ test was applied for categorical variables to assess significant differences between the groups.

To determine the association between significant determinants identified in the bivariate analysis and frailty, a hierarchical logistic regression analysis was performed. The determinants were entered sequentially based on their importance until all relevant variables were included. The results of this regression are reported as odds ratios (OR) with 95% confidence intervals (CI), providing insights into the strength and direction of associations between frailty and its potential risk factors. All statistical analyses were performed using SPSS version 20 (IBM Corp., IBM SPSS Statistics for Windows, Version 20.0. Armonk, NY: IBM Corp; 2011.). A two‐sided *p* < 0.05 was considered a statistically significant level.

## 3 Results

In this study, data from 258 elderly individuals were analyzed. The mean age of the participants was 66 ± 5.56 years. The age range of the participants varied between 60 and 86 years. The average number of medications taken by elderly people was 3.43 ± 2.29.

Of the elderly people, 32.9% had three children, representing the highest frequency among the participants. The frequency distribution of other demographic variables is presented in Table 1.

**Table 1:** Demographic characteristics of study participants.

Variable	Variable levels	Frequency (%)
Age	60–70 years	200 (77.5)
	70–80 years	54 (20.9)
	> 80	4 (1.6)
Sex	Male	116 (45)
	Female	142 (55)
Marriage	Marriage	229 (88.8)
	Single	29 (11.2)
Education	Secondary school	21 (8.1)
	Diploma (12‐year formal education)	64 (24.8)
	Associate degree	60 (23.3)
	Bachelor's degree	94 (36.4)
	MSc	19 (7.4)
Housing situation	Owner house	226 (87.6)
	Tenant	32 (12.4)
Arrangement of life	Single	38 (14.7)
	With wife or husband	86 (33.3)
	With children and wife or husband	134 (51.9)
Number of children	0	34 (13.2)
	3–1	159 (61.6)
	6–4	63 (24.4)
	6 >	2 (0.8)
Polypharmacy	Yes	88 (34.1)
	No	170 (65.9)
Quality of sleep	Good	182 (70.5)
	Poor	76 (29.5)

The mean frailty score based on the Edmonton Frail Scale was 3.44 ± 2.16. Frailty had a significant relationship with the variables of age, education, polypharmacy, and poor sleep quality. However, there was no statistically significant relationship between frailty and other variables such as gender, marital status, housing status, living arrangements, and the number of children. These findings are shown in Table 2.

**Table 2:** The bivariate relationship between potential risk factors and frailty.

Variable		Frailty		*p* value^a^
		Yes	No	
		45 (17.4)	213 (82.6)	
Age		68.20 ± 6.03	65.91 ± 5.41	0.01
Sex	Male	17 (37.8%)	99 (46.5%)	0.27
	Female	28 (62.2%)	114 (53.5%)	
Marriage	Marriage	39 (86.7%)	190 (89.2%)	0.63
	Single	6 (13.3%)	23 (10.8%)	
Education	Secondary school	9 (20%)	12 (5.6%)	< 0.001
	Diploma (12‐year formal education)	14 (31.1%)	50 (23.5%)	
	Associate degree	14 (31.1%)	46 (21.6%)	
	Bachelor's degree	7 (15.6%)	7 (40.8%)	
	MSc	1 (2.2%)	18 (8.5%)	
Housing situation	Owner house	39 (86.7%)	187 (87.8%)	0.84
	Tenant	6 (13.3%)	26 (12.2%)	
Arrangement of life	Single	9 (20%)	29 (13.6%)	0.31
	With wife or husband	17 (37.8%)	69 (32.4%)	
	With children and wife or husband	19 (42.2%)	115 (54%)	
Number of children		2.6 ± 1.49	2.6 ± 1.41	0.13
Polypharmacy	Yes	30 (66.7%)	58 (27.2%)	< 0.001
	No			
		15 (33.3%)	155 (72.8%)	
Quality of sleep	Good	25 (55.6%)	157 (73.7%)	0.02
	Poor	20 (44.4%)	56 (26.3%)	

^a^ Continuous and categorical variables are reported as mean ± standard deviation and frequency (percentage) and compared between two groups using independent *t*‐test and *χ* ^2^, respectively.

In this study, 34.1% of elderly individuals were exposed to polypharmacy, defined as taking five or more medications. To assess the association between potential determinants and frailty, we conducted a hierarchical logistic regression analysis. Variables were entered sequentially into the model based on their relevance until all significant predictors were included. The results are presented as *B* values, odds ratios (OR), and 95% confidence intervals (CI), indicating the strength and direction of associations. These findings are shown in Table 3 below.

**Table 3:** Hierarchical logistic regression for the association of the potential determinants of frailty.

Predictors			Model*			
			1	2	3	4
Polypharmacy	Yes		5.34 (2.68–10.6)	4.87 (2.41–9.82)	5.20 (2.52–10.74)	6.16 (2.8–13.50)
	No (reference)	1				
Quality of sleep	Good			1.64 (0.80–3.31)	1.82 (0.88–3.78)	1.45 (0.67–3.15)
	Poor (reference)	1				
Age					1.09 (1.02–1.16)	1.07 (1.01–1.15)
Education	Secondary school (reference)	1				
	Diploma (12‐year formal education)					0.421 (0.129–1.37)
	Associate degree					0.253 (0.07–0.84)
	Bachelor's degree					0.10 (0.02–0.37)
	MSc^a^					0.07 (0.00–0.74)

*Data are presented as OR (95% CI).

Model 1: polypharmacy as the primary independent variable.

Model 2: adjusted for polypharmacy and quality of sleep.

Model 3: further adjusted for age in addition to polypharmacy and quality of sleep.

Model 4 (final model): Adjusted for polypharmacy, quality of sleep, age, and education level.

^a^ Master of Science.

### 3.1 Model 1

The first model examined the impact of polypharmacy on frailty. Compared to individuals without polypharmacy, those with polypharmacy exhibited a significantly increased odds of frailty (*B* = 1.67, OR = 5.34, 95% CI: 2.68–10.6), highlighting polypharmacy as a strong risk factor.

### 3.2 Model 2

In the second model, we adjusted for the quality of sleep. While polypharmacy remained significantly associated with frailty (*B *= 1.58, OR = 4.87, 95% CI: 2.41–9.82), poor sleep quality was also introduced into the model. However, the association between sleep quality and frailty was not statistically significant (*B* = 0.49, OR = 1.64, 95% CI: 0.80–3.31).

### 3.3 Model 3

The third model incorporated age as an additional determinant. Polypharmacy remained a significant predictor (*B* = 1.64, OR = 5.20, 95% CI: 2.52–10.74), while poor sleep quality remained nonsignificant (*B* = 0.60, OR = 1.82, 95% CI: 0.88–3.78). However, age emerged as a significant factor (*B* = 0.07, OR = 1.09, 95% CI: 1.02–1.16), suggesting that older age is associated with a higher likelihood of frailty.

### 3.4 Model 4 (Final Model)

In the final model, education level was added as a potential determinant. Polypharmacy remained a strong predictor (*B* = 1.82, OR = 6.16, 95% CI: 2.8–13.50), while age continued to show a significant association with frailty (*B* = 0.06, OR = 1.07, 95% CI: 1.01–1.15). Education level was also found to be significantly associated with frailty. Compared to individuals with only secondary education, those with higher levels of education exhibited lower odds of frailty (*B* = −2.66, OR for MSc = 0.07, 95% CI: 0.00–0.74).

## 4 Discussion

This study examined the prevalence of frailty and its related factors among retired elderly individuals (60 years and older) in the Isfahan Education Retirees Association. According to the findings, 17.4% of the elderly participants exhibited varying degrees of frailty. This indicates that nearly one in six elderly individuals in the Iranian community may be frail. The prevalence of frailty in Iranian studies has been reported variably. For example, in the study by Amrahi Tabieh and colleagues in Tabriz, the prevalence of frailty was reported to be 30.3%. In the study by Jafarian and colleagues, the Tilburg Frailty Indicator was used, and 62.5% of the elderly were identified as frail. In the study by Hosseini, among hospitalized elderly individuals, frailty prevalence was reported to be 40.4%. The prevalence of frailty in all these studies was higher than in our study [18, 34, 35]. However, the prevalence of frailty in the study by Saeidimehr and colleagues was reported as 10.4%, which was lower than our study. In that study, data were collected based on the Deficit Accumulation Frailty Index [36]. Differences in the reported prevalence of frailty across various studies largely depend on the population being studied, the measurement tools used to assess frailty, and the impact of demographic factors on elderly people.

The results of this study confirmed that frailty and disability are more prevalent among older adults aged 70 years and older than in the compared to other elderly people studied. This finding has been corroborated by other studies [18, 37]. Due to the aging process, a gradual decline in physiological reserves and functioning across multiple body systems leads to frailty [7].

The findings of this study also confirmed the relationship between lower education levels and a higher prevalence of frailty among elderly Iranian people. Several similar studies have also verified the association between frailty and lower educational attainment among elderly people [38, 39]. Lower education levels may contribute to frailty by creating difficulties for elderly people to access healthcare services. It influences lifestyle choices, too. Additionally, individuals' education levels may significantly impact cognitive function, serving as a key factor in preventing frailty. Education is a social determinant, and recent studies have shown that social factors play a role in the development of frailty [40]. Therefore, identifying this factor and its potential role in the pathophysiology of frailty is of great importance and warrants further exploration in future research.

A significant association was found between poor sleep quality and frailty. Amrahi Tabieh and colleagues in Iran also reported a relationship between poor sleep quality and frailty [18]. Similarly, Hyo Sun and colleagues reported that poor sleep quality was associated with higher levels of frailty among elderly people [41].

Several potential mechanisms may explain the relationship between sleep disorders and frailty:

1. Sleep disorders may contribute to increased oxidative stress and metabolic changes towards catabolism, leading to a cumulative risk of frailty [15].
2. Sleep disturbances may reduce growth hormones, IGF‐1 growth factor, and sex hormones, including testosterone, which can lead to increased sarcopenia and thereby contribute to frailty [42].
3. Circadian rhythm disruptions caused by sleep disorders may impair immune system regulation by increasing systemic inflammatory factors, which play a role in the development of frailty [43].

The findings of this study also indicated that elderly individuals who took more medications were more vulnerable to frailty. In a systematic review [21] that examined the relationship between polypharmacy and frailty, having multiple chronic diseases simultaneously was identified as a contributing factor to the consumption of five or more medications, leading to a higher prevalence of frailty. Furthermore, taking more medications increases the risk of other adverse drug effects, such as drug–drug interactions, potentially inappropriate medication prescribing, or adverse drug reactions, which in turn contribute to the higher prevalence of frailty among elderly people. There also appears to be a strong link between frailty and changes in pharmacokinetic responses, particularly in metabolism and excretion, relative to an individual's chronological age [44].

## 5 Conclusion

Considering that frailty in old age can lead to adverse medical, economic, and social outcomes and affect the quality of life for elderly people, there is a need for more serious planning and further studies in this area. Various occurrences during old age and related demographic factors may account for differences in reported frailty across different studies. In this study, frailty was reported among the elderly people with advanced age (> 70 years), lower education levels, a history of poor sleep quality, and those with a history of polypharmacy. Thus, these groups of elderly people require increased care services and timely interventions to reduce frailty‐related complications. Moreover, to identify and design timely interventions, early screening and diagnosis should be conducted among younger elderly individuals to prevent progression to later stages of frailty. Given that this study is among the first to examine the prevalence and associated factors of frailty among Iranian retirees, more comprehensive studies incorporating behavioral factors are needed to improve the precision of service delivery to elderly people.

## Author Contributions

**Majid Rahimi:** writing – original draft, writing – review and editing, methodology, conceptualization, validation, resources. **Maryam Shirdozham:** conceptualization, project administration, funding acquisition, investigation, writing – original draft, supervision. **Awat Feizi:** methodology, software, data curation, formal analysis, validation.

## Ethics Statement

The study received approval from the Isfahan University of Medical Sciences Ethics Committee (Research Proposal Code: 1401336 and Ethical Code: IR. MUI. RESEARCH. REC.1402.055). Informed consent was obtained from all study participants. All steps of the study were conducted in strict adherence to the ethical standards of the Helsinki Declaration. We also followed the CONSORT guidelines (http://www.consort-statement.org/) for reporting clinical trials, ensuring that all relevant guidelines and regulations were followed in all methods.

## Consent

After explaining the purpose and the nature of the study, informed consent was obtained from the participants, and they had the right to refuse to fill it out. Anonymity and confidentiality of participants were ensured by the authors.

## Conflicts of Interest

The authors declare no conflicts of interest.

## Transparency Statement

The corresponding author, Maryam Shirdozham, affirms that this manuscript is an honest, accurate, and transparent account of the study being reported; that no important aspects of the study have been omitted; and that any discrepancies from the study as planned (and, if relevant, registered) have been explained.

## Data Availability

The data sets used and/or analyzed during this study are available from the corresponding author.
